# Decreased Right Prefrontal Synchronization Strength and Asymmetry During Joint Attention in the Left-Behind Children: A Functional Near-Infrared Spectroscopy Study

**DOI:** 10.3389/fphys.2021.759788

**Published:** 2021-11-11

**Authors:** Keya Ding, Hongan Wang, Chuanjiang Li, Fulin Liu, Dongchuan Yu

**Affiliations:** ^1^Key Laboratory of Child Development and Learning Science, Research Center for Learning Science, Southeast University, Nanjing, China; ^2^School of Biological Science and Medical Engineering, Southeast University, Nanjing, China; ^3^Hangzhou College of Early Childhood Teachers’ Education, Zhejiang Normal University, Hangzhou, China; ^4^Department of Child Development and Behavior, Third Affiliated Hospital of Zhengzhou University, Zhengzhou, China

**Keywords:** left-behind children in rural area, synchronization, right prefrontal cortex, social cognition and interaction, fNIRS (functional near-infrared spectroscopy), S-Renyi synchronization estimation

## Abstract

Although there are millions of left-behind children in China, the researches on brain structure and functions in left-behind children are not sufficient at the brain imaging level. This study aimed to explore whether there is decreased prefrontal synchronization during joint attention in left-behind children. Sixty children (65.12 ± 6.54 months, 29 males) with 34 left-behind children were recruited. The functional near-infrared spectroscopy (fNIRS) imaging data from the prefrontal cortex during joint attention, as well as behavioral measures (associated with family income, intelligence, language, and social-emotional abilities), were collected. Results verified that brain imaging data and behavioral measures are correlative and support that left-behind children have deficits in social-emotional abilities. More importantly, left-behind children showed decreased synchronization strength and asymmetry in the right middle frontal gyrus during joint attention. The findings suggest that decreased right prefrontal synchronization strength and asymmetry during joint attention might be vulnerability factors in the development of left-behind children.

## Introduction

The rapid urbanization has caused the emergence of a special group of left-behind children with deprivation of parental care [being considered as an early-life stress (Mueller et al., 2010)] in China. Previous studies have confirmed that left-behind children have lower academic achievement, emotional and behavioral problems, serious mental health problems (He et al., 2012; Fellmeth et al., 2018), and even psychological disorders (Tomşa and Jenaro, 2015). More seriously, the successive deprivation of parental care might contribute to potential and irreversible risks for these left-behind children across the lifespan. On the other hand, by 2018, there were 6.97 million left-behind children in rural areas in China (Ministry of Civil Affairs of China, 2021), of which the largest population (more than 50%) aged between 6 and 13 years old. The emergence of such a huge number of left-behind children might initiate serious economic, social, and even political issues in the future in China.

Deprivation of parental care may usually lead to physical or/and emotional neglect in left-behind children (Mueller et al., 2010; Teicher et al., 2016; McLaughlin et al., 2017), and thereby be considered as a kind of adverse childhood experiences (McLaughlin et al., 2014, 2017; Teicher et al., 2016). It has been hypothesized and widely accepted (Teicher et al., 2016; McLaughlin et al., 2017) that early experiences are crucial in shaping individuals’ brain structure and functions. Therefore, it is not surprising that early caregiver deprivation (especially early emotional neglect) contributes to unexpected abnormal changes in brain structure, function, connectivity, and activation pattern [see (Teicher et al., 2016) for a review], such as a general reduction in cortical thickness (particularly in the prefrontal cortex) (McLaughlin et al., 2014, 2017), accelerated development of the connectivity between the amygdala and medial prefrontal cortex (Gee et al., 2013), a more widespread activation pattern of dorsolateral prefrontal cortex during trials requiring inhibitory control (Mueller et al., 2010), and decreased oxygenated blood flow to the medial prefrontal and orbitofrontal cortices during measures of decision making (Mark, 2019). In the meanwhile, early caregiver deprivation also contributes to children’s deficits associated with cognitive, emotional, and social functions (Gee et al., 2013; McLaughlin et al., 2014, 2017; Teicher et al., 2016; Mark, 2019). In addition, both animal experiments and longitudinal studies of orphans have verified that early caregiver deprivation may initiate deterioration of emotional and cognitive processes in adulthood (Chugani et al., 2001; Mueller et al., 2010; Marco et al., 2015).

Although there are so many left-behind children in China, the researches on left-behind children are not sufficient. Furthermore, most studies are at the behavioral level. However, little is known whether there are brain structural or functional alterations (or even impairments) in left-behind children, compared to their counterparts (non-left-behind children). Thus far, as far as we know, there are only two brain imaging studies on left-behind children, aimed to reveal the brain structural or functional alterations. Zhao et al. (2016), for instance, reported that left-behind children have weak network efficiency in the resting-state functional magnetic resonance imaging (fMRI) brain network (Zhao et al., 2016). This abnormal feature was expected to interpret the influence of parent-child separation on left-behind children. Another research also found the presence of altered brain structure (e.g., gray matter volume and fractional anisotropy) in left-behind children (Fu et al., 2019). Remarkably, both studies investigated only the resting-state brain structure and network (task-free). The current study aimed to analyze the task-related brain imaging data and bring new insights into the understanding of brain structural and functional changes in left-behind children.

Joint attention refers to the coordinated focus of attention between two or more individuals on a common partner or event, and can be classified into two categories, i.e., Responding to Joint Attention (RJA) – following the direction of the gaze and gestures of others, and Initiating Joint Attention (IJA) – sharing individuals’ motivations and intentions with others. Joint attention involves aspects of executive attention regulation, inhibitory control, and self-monitoring that are critical for the subsequent development of social skills (Mundy and Acra, 2006; Mundy and Newell, 2007). Therefore, developing RJA and IJA skills in infancy is thought to be crucial for language development, general learning, and social functions across the lifespan (Baldwin, 1995; Tomasello, 1995). Individuals’ differences in joint attention are highly related to long-term parental social responsiveness and the frequency and intensity of sharing experiences with others. The impairment of joint attention may contribute to social dysfunctions (Dawson et al., 2002). A particularly extreme example is autism, characterized by the lack of spontaneous sharing experiences with others (Charman, 2003). The current study aimed to utilize joint attention tasks as the stimuli of task-related brain imaging data for left-behind children.

Synchronization between neural signals refers to the temporal coordination of neural oscillatory activities in a parallel manner (Pikovsky et al., 2001). Synchronization of neural signals is a hypothetical self-organization model and mechanism of functional connectivity involved in the information processing in the nervous systems. These technical advantages of synchronization have actually promoted its utility in the understanding of human brain dynamics measured by neuroimaging (Ramon et al., 2017). The impairment of synchronization contributes to several neurological symptoms (Hogan et al., 2003; Spencer et al., 2003; Just et al., 2004; Koenig et al., 2005; Jalili et al., 2007; Dinstein et al., 2011; Querne et al., 2017). For instance, disrupted anti-phase synchronization between default-mode and task-positive networks is considered as a neuropathological basis of Attention Deficit Hyperactivity Disorder (ADHD) (Querne et al., 2017). Patients with autism exhibit weaker synchronization across various brain regions (including language areas) compared to healthy controls, and their severity of autism may be predicted and identified by the strength of synchronization (Just et al., 2004; Dinstein et al., 2011). Schizophrenia patients show the presence of bilaterally increased synchronization among temporal regions and decreased synchronization over the postcentral/parietal regions neighboring the midline (Spencer et al., 2003; Jalili et al., 2007). Alzheimer’s disease is often associated with loss of local and global synchronous activity between brain regions (Hogan et al., 2003; Koenig et al., 2005). On the other hand, the prefrontal cortex engages in various cognitive functions such as inhibition, working memory, and cognitive flexibility. Through its inhibitory pathway to the amygdala, the prefrontal cortex involves in emotional cognition and regulation. To summarize, disrupted neural synchronization in the prefrontal cortex appears to be a notable characteristic of neurodevelopmental disorder that is evident at very early stages of development. This finding supports that there is decreased prefrontal synchronization in left-behind children, compared to their counterparts (non-left-behind children). To test this hypothesis became the main motivation of the current study.

Taken together, this study aimed to detect the hypothesis of decreased prefrontal synchronization during joint attention in left-behind children, by the functional near-infrared spectroscopy (fNIRS) due to its safety, portability, relatively high temporal resolution, insensitivity to head movement, and reliable clinical utility (Ferrari and Quaresima, 2012; Mark, 2019). It was expected to uncover the differences of prefrontal synchronization strength and asymmetry during joint attention between left-behind and non-left-behind children. Behavioral measures (associated with family income, intelligence, language, and social-emotional abilities) were collected. This study also discussed the correlation between behavioral measures and prefrontal synchronization strength and asymmetry during joint attention.

## Materials and Methods

### Procedure

All study procedures and research methods were carried out in accordance with the Declaration of Helsinki (1964) by the World Medical Association concerning human experimentation and were approved by the Research Ethics Committee at Southeast University. Online informed consent was obtained from all caregivers and oral consent was obtained from all participated children. All participants were recruited from 6 classrooms of a kindergarten in a rural village of Guangxi province, China. The interested families contacted the teachers to confirm their understanding of the research content and completed a brief screening to determine eligibility. Children were excluded from participants in this study if they had a history of a neurological disorder, loss of consciousness, sensory impairments, autism spectrum disorder, or an intellectual disability. Left-behind children were recruited if one of their parents or both parents had been gone for over 3 months (UNICEF, 2009). Each child received an age-appropriate toy after completing the study.

### Participants

Although 82 children were recruited in the study, 8 subjects were excluded due to loss of fNIRS data, unacceptable head movement, or refusal to attend the experiments. Fourteen children were further excluded due to poor data quality after pre-processing. In order to ensure the reliability of left-behind children’s information, participants’ teachers and parents were asked to report the lasting time and status of parent-child separation simultaneously. A participant was taken as a “left-behind” subject only when both reports were highly consistent. By this way, only 60 participants (26 males) aged 52–76 months (65.12 ± 6.54 months) without gender difference (χ^2^ = 2.56, *p* = 0.11) were actually considered in this study, including 34 left-behind children and 26 non-left-behind children.

### Family Income

Participants’ parents were required to report their family monthly income, according to the following coding rules: (i) “1” represents a monthly income of 2,000 RMB or less; (ii) “2” represents an income of 2,000 to 4,000 RMB; (iii) “3” represents an income of 4,000 to 6,000 RMB; (iv) “4” represents an income of 6,000 to 8,000 RMB; and (v) “5” represents an income of 8,000 RMB or more.

### Behavioral Tasks

Participants were required to complete an intelligence test and a Chinese receptive language task by the Combined Raven Test (CRT) (Wang and Qian, 2007) and the Peabody Picture Vocabulary Test-Revised (PPVT) (Lu and Liu, 1998), respectively. In addition, participants were instructed to conduct two tablet-based tasks, namely the Facial Expression Recognition Task (FERT) and the Emotional Comprehension Task (ECT), to assess their social-emotional abilities to distinguish facial emotions and recognize expressions (feelings), respectively. In the FERT task, participants were asked to select one correct facial emotion from 4 candidates according to audio instructions (10 cues), where all facial emotions were chosen from the Mind Reading database (Baron-Cohen et al., 2004). The ECT task consisted of 10 daily life scenarios, where the main character in each scenario had different expressions. Participants were instructed to select the most appropriate expression of the main character from 4 candidates. In combination with accuracy and reaction time, this study suggested a comprehensive index (CI) to measure individuals’ social-emotional abilities, which was defined by


(1) $${\text{CI}}^{\left(i\right)}=5*{\text{Accuracy}}^{\left(i\right)}+5*\frac{l\,o\,g\,6000-l\,o\,g\,\mathrm{Reaction}\,{\mathrm{Time}}^{\left(i\right)}}{l\,o\,g\,6000-l\,o\,g\,300}$$

where *i* = 1 and *i* = 2 represent the case of ECT and FERT, respectively. The value of CI^(*i*)^ may be ranged from 0 to 10. The higher the accuracy and the shorter the reaction time, the higher the value of CI^(*i*)^.

### Joint Attention Tasks

**FIGURE 1:**
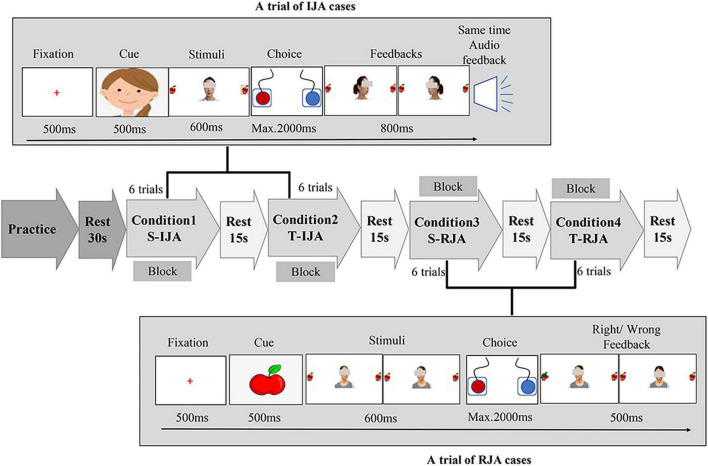
Joint attention task schematic. The experiment consisted of two joint attention cases (i.e., IJA and RJA) under two kinds of social partners (i.e., stranger and teacher), and thereby was a 2 × 2 task (stranger/teacher × IJA/RJA) that actually included four conditions, i.e., condition 1 (S-IJA, corresponding to Stranger-IJA), condition 2 (T-IJA, corresponding to Teacher-IJA), condition 3 (S-RJA, corresponding to Stranger-RJA), and condition 4 (T-RJA, corresponding to Teacher-RJA). Each condition was a block that included 6 trials and would be repeated three times in the experiment. In the RJA trial (see the bottom of the figure), participants were instructed to follow a partner’s (stranger’s/teacher’s) gaze turned leftward or rightward via pressing the left or right button as quickly and accurately as possible. While, in the IJA trial (see the top of the figure), participants were asked to freely choose to shift the gaze of the interacting partner (teacher/stranger) by pressing the left or right button. The partner (teacher/stranger) shifted his/her gaze leftward or rightward dependent on the participant’s choice.

The current study expected to investigate the difference between social interaction with familiar or unfamiliar partners in two joint attention tasks (i.e., IJA and RJA cases). For this purpose, a modified version (see Figure 1 for detailed information) of the joint attention experimental paradigm (Oberwelland et al., 2006) was proposed, for which only two target directions (i.e., leftward and rightward) were considered, the “cheeses” were substituted by the “apples,” and visual and verbal feedbacks to participants were added. The photographs from both participant’s close teacher and a female stranger were utilized to imitate the social interaction with familiar or unfamiliar partners, respectively. These photographs were gathered according to the following rules. For each teacher or stranger, five photographs were taken, for which three of them were with the gaze turned forward, leftward, rightward, while two of them were happy and sad faces used as feedbacks to correct or incorrect responses. Guiding by an on-screen cue (an apple or a cartoon person’s face), each participant was required to initiate or respond to a partner (stranger/teacher) by conducting a 2 × 2 conditioned task (stranger/teacher × IJA/RJA).

#### Responding to Joint Attention Task

For an RJA trial, a cartoon person’s face was present in the midline of the computer screen for 500 ms. Then, participants were instructed to follow a partner’s (stranger’s/teacher’s) gaze turned leftward or rightward (600 ms) via pressing the left or right button (maximum of 2,000 ms) as quickly and accurately as possible. At the end of each interaction, participants received a 500 ms feedback (a happy or sad face, corresponding to correct or incorrect response, respectively). After that, a red cross (fixation) was present for 500 ms before the next trial. Each block (i.e., S-RJA (Stranger-RJA) or T-RJA (Teacher-RJA) condition) included 6 trials and was repeated three times in the experiment.

#### Initiating Joint Attention Task

For an IJA trial, an apple picture was present in the middle of the computer screen for 500 ms. Then, a photography of the interactive partner (teacher/stranger) was present in the midline, with two apples locating on the left and right side. Different from the RJA task, the interactive partner (teacher/stranger) in the IJA task was looking forward (toward the participant), neither leftward nor rightward. The participant was asked to freely choose to shift the gaze of the interacting partner (teacher/stranger) by pressing the left or right button (maximum of 2,000 ms). Dependent on the participant’s choice, the partner (teacher/stranger) shifted his/her gaze leftward or rightward. Meanwhile, the participant received verbal feedback “Yeah!,” indicating that the partner (teacher/stranger) followed the participant’s gazing. Each block [i.e., S-IJA (Stranger-IJA) or T-IJA (Teacher-IJA) condition] included 6 trials and was repeated three times in the experiment.

The joint attention tasks with a block design were programmed and conducted with the software E-prime (Psychology Software Tools Inc., Pittsburgh, PA, United States, Version 1.0). The whole task actually included four experimental conditions, i.e., condition 1 (S-IJA), condition 2 (T-IJA), condition 3 (S-RJA), and condition 4 (T-RJA). The experimental protocol with a total of 12 blocks (6 trials for each block) was clearly shown in Figure 1, where each block was corresponding to a condition and each condition included three blocks. The whole experiment (lasting 7–8 min) was presented on a 14-inch computer screen (resolution 1024^∗^768) with a white background. Before a formal experiment, participants were instructed to do some practice for the understanding of the whole experimental procedure under the standard of exceeding 80% accuracy.

### Functional Near-Infrared Spectroscopy Acquisition and Processing

**FIGURE 2:**
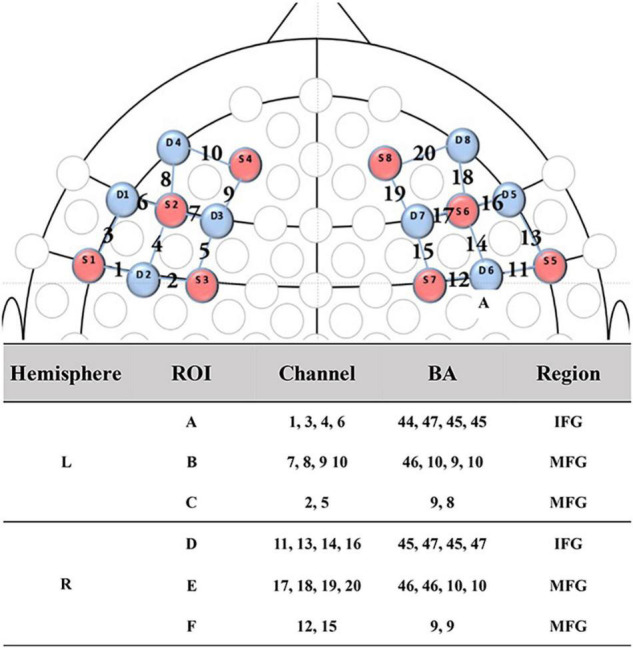
fNIRS channel and probe configuration. The 2D map illustrated the distribution of probes (red and blue representing the source and detector probe, respectively) and 20 channels. While, the table showed the ROIs, details of channels, brain regions and correspondent Brodmann area. ROI, regions of interest; IFG, inferior frontal gyrus; MFG, middle frontal gyrus; BA, Brodmann area.

During joint attention, the oxy-hemoglobin (HbO) and deoxy-hemoglobin (HbR) concentration changes at the wavelengths of 630 and 850 mm were recorded using an fNIRS equipment, i.e., NIRSport 8^∗^8 (NIRx Medical Technology LLC, Glen Head, NY, United States) Optical Topography System, with sampling frequency 7.81 Hz. This fNIRS equipment had 8 light sources and 8 detectors, evenly distributed over the left and right prefrontal regions according to the 10–10 transcranial positioning system. Hence, this fNIRS equipment actually had 20 neural channels covering bilateral inferior frontal gyrus (IFG) and middle frontal gyrus (MFG) (see Figure 2). For better analysis, these 20 channels were divided into 6 regions (A to F) of interest, where Region A contained channels 1, 3, 4, and 6; Region B contained channels 7, 8, 9, and 10; Region C contained channels 2 and 5; Region D contained channels 11, 13, 14, and 16; Region E contained channels 17, 18, 19 and 20; Region F contained channels 12 and 15. It is clear that Regions D, E, and F were located in the right prefrontal lobe; and Regions A, B, and C were located in the left prefrontal lobe. Regions D-A, E-B, and F-C were taken as region pairs in the evaluation of asymmetry.

The fNIRS data were preprocessed by the Homer2 toolbox (Huppert et al., 2009). After raw intensity data being converted to optical density units, checking and repairing steps were conducted by the channel artifact detection and spline correction method, in order to ensure data quality even in the presence of large artificial signal variations (Scholkmann et al., 2010). Then, high- and low-frequency noise of signals was removed by a bandpass filter with cutoff frequencies at 0.01–0.2 Hz. Finally, hemoglobin concentration variations were calculated by a modified Beer-Lambert law (Delpy et al., 1988).

This study considered HbO concentration only, due to its high sensitivity to changes in regional cerebral blood flow (Strangman et al., 2002). In addition, the time window of interest was set at the range of 11–21 s for each condition (block). Therefore, HbO concentration changes at the range of 11–21 s were actually taken into account for time-series analysis.

### Synchrony Measurement and Asymmetry

Let $\text{X}=\left\{x_{i}^{t}\right\}$ be a multivariate measurement consisting of *N* time-series of channels, regions, or even the whole brain network, where *t* = 1,2,,*L*; *i* = 1,2,,*N*; $x_{i}^{t}$ represents the observation vector of the *t-th* sample of the *i-th* channel/region. Among others, S-estimator were widely used and showed its clinical utility in the evaluation of synchronization strength. The current study suggested a modified S-estimator version using Renyi entropy, called S-Renyi sync-estimator (Biey and Righero, 2009), for the evaluation of synchronization strength. The advantage of S-Renyi method was based on its stronger robustness and lower noise sensitivity, compared to the original version of S-estimator (Cai et al., 2019). The S-Renyi sync-estimator was defined by:


(2) $$\text{SRenyi}=1+\frac{1}{\left(\alpha -1\right)\,\text{log}\,\left(N\right)}\,l\,o\,g\,\sum_{i=1}^{N}{\left(r_{i}/N\right)}^{\alpha }$$

where *r_i_* represents the eigenvalue of the correlation matrix **R** of **X**. The value of S-Renyi can be regulated by the parameter α : (i) When α tends to 1, Renyi entropy tends to Shannon entropy; and (ii) When α = 6, SRenyi of two time-series has the most similar performance with simple Pearson’s correlation (Cai et al., 2019).

The synchronization asymmetry index (SAI) was proposed to investigate the synchronization strength difference between a region/hemisphere and its contralateral region/hemisphere, which was defined as follows (Johnstone et al., 2007):


(3) $$\mathrm{SAI}\,\left(x-y\right)=\frac{S\,R\,e\,n\,y\,i^{\left(x\right)}-S\,R\,e\,n\,y\,i^{\left(y\right)}}{S\,R\,e\,n\,y\,i^{\left(x\right)}+S\,R\,e\,n\,y\,i^{\left(y\right)}}$$

where *SRenyi*^(*x*)^ and *SRenyi*^(*y*)^ represent the synchronization strength of region *x* and its contralateral region *y*, respectively. Positive or negative value of *SAI*(*x*−*y*) implies right and left asymmetry, respectively.

### Statistical Analysis

The experiments in this study actually included three factors, i.e., task-types (IJA and RJA tasks), partner-types (teacher and stranger), and separation-types (left-behind and non-left-behind children). By SPSS (IBM Corporation, Armonk, NY, United States), a three-factor MANOVA (with 2 task-types^∗^2 partner-types^∗^2 separation-types) was conducted to examine the main effects for synchronization strength and asymmetry results, with the significance level 0.05. Correlations between behavioral measures and synchronization strength and asymmetry were calculated by Pearson’s correlation with R language (version 4.0.2).

## Results

### Behavioral Performance

Table 1 summarized our results and showed that: (i) There were no statistical differences between left-behind and non-left-behind children in age, language (PPVT test), intelligence (Combined Raven Test), and family income (*p’s* > 0.05); (ii) There were no statistical difference between left-behind and non-left-behind children in the measurement of the Facial Expression Recognition Task (*p* > 0.05); and (iii) Non-left-behind children have higher comprehensive index (*t* = 2.30, *p* = 0.025) in the Emotional Comprehension Task.

**TABLE 1:** Demographic characteristics and differences of behavioral measures between left-behind and non-left-behind children.

	**Non-left-behind children**	**Left-behind children**	***t***	***p***
Age (month)	64.685.70	65.437.15	–0.45	0.65
PPVT	44.3213.37	48.9118.26	–1.12	0.27
Combined raven test	18.124.53	17.484.13	0.55	0.58
Family income	2.691.41	3.351.12	–1.96	0.06
CI ^(1)^	4.371.06	3.741.03	2.30	0.025^*^
CI ^(2)^	5.461.10	5.261.14	0.68	0.50

PPVT, Peabody Picture Vocabulary Test; CI ^(1)^, comprehensive index of the Emotional Comprehension Task; CI ^(2)^, comprehensive index of the Facial Expression Recognition Task, *p < 0.05.

### Synchronization and Asymmetry

**FIGURE 3:**
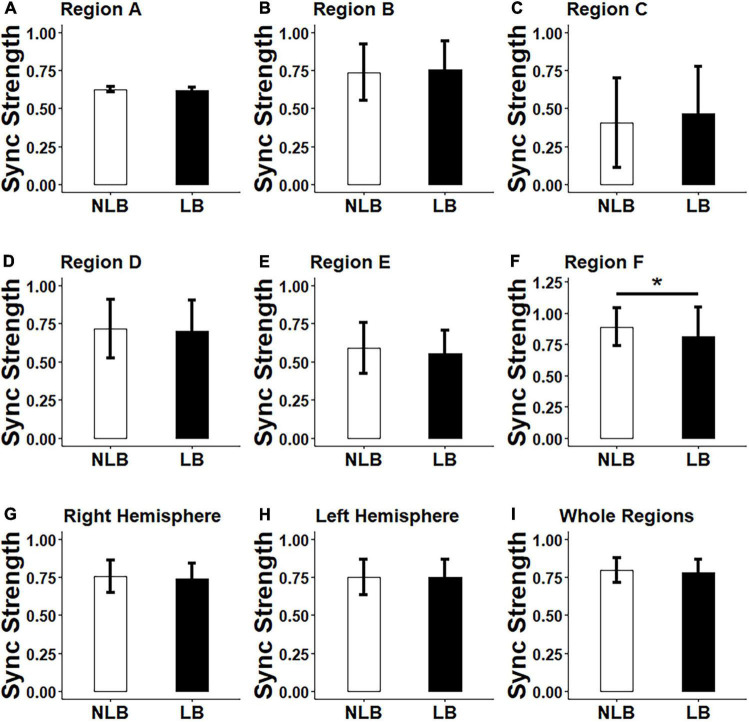
Differences between left-behind and non-left-behind children in synchronization strength of **(A–F)**: Regions A–F, **(G–H)**: Right and left hemispheres, and **(I)**: Whole regions. NLB, non-left-behind; LB, left-behind; Sync, synchronization; *p < 0.05.

**FIGURE 4:**
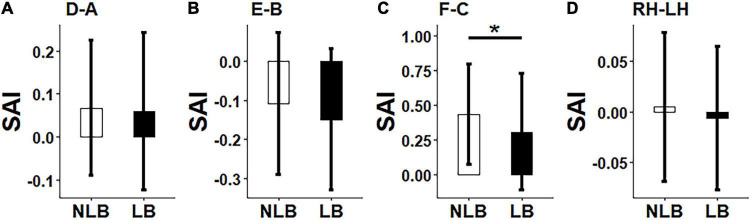
Differences between left-behind and non-left-behind children in synchronization asymmetry of **(A)**: Region-pair D-A; **(B)** Region-pair E-B, **(C)** Region-pair F-C, and **(D)** Hemisphere-pair RH-LH. NLB, non-left-behind; LB, left-behind, *p < 0.05.

A three-factor MANOVA was conducted on synchronization strength and asymmetry results, where three factors included task-types (IJA and RJA tasks), partner-types (teacher and stranger), and separation-types (left-behind and non-left- behind children). The statistical results (Figures 3, 4) showed that: (i) Non-left-behind children had significantly higher synchronization strength of Region F than left-behind children (*F* = 5.051, *p* = 0.028); (ii) Non-left-behind children had significantly higher synchronization asymmetry in region-pair F-C than left-behind children (*F* = 5.96, *p* = 0.018); (iii) The synchronization asymmetry between left and right hemispheres in left-behind and non-left-behind children was influenced by the partner-types (teacher and stranger) and task-types (RJA and IJA) (*F* = 4.166, *p* = 0.046); and (iv) For synchronization strength in Region E, there was a significant interaction effect among the three factors (*F* = 6.185, *p* = 0.016).

By *post hoc* tests, it was further found that: (i) When left-behind children interacted with teachers, RJA evoked higher synchronization asymmetry between left and right hemispheres than IJA (*p* = 0.015); (ii) For left-behind children in the RJA task, the interaction of child-teacher evokes higher synchronization asymmetry between left and right hemispheres than that of child-stranger (*p* = 0.019); (iii) When interacted with strangers in the IJA task, non-left-behind children evokes higher synchronization asymmetry between left and right hemispheres than left-behind children (*p* = 0.046); (iv) When non-left-behind children interacted with strangers, the RJA task evoked higher synchronization in Region E than the IJA task (*p* = 0.046); (v) For left-behind children in the RJA task, the interaction of child-teacher evokes higher synchronization in Region E than that of child-stranger (*p* = 0.019); (vi) When interacted with strangers in the RJA task, non-left-behind children evokes higher synchronization in Region E than left-behind children (*p* = 0.005); and (vii) In addition to above results, no statistical differences had been found for synchronization strength and asymmetry in other brain regions, left and right hemispheres or the whole regions (*p*’s > 0.05).

### Correlation Analysis

The experiments included four conditions, i.e., S-IJA, S-RJA, T-IJA, and T-RJA. For each condition, we calculated the correlations between behavioral measures (including family income, intelligence, language, and social-emotional abilities) and synchronization strength as well as synchronization asymmetry in different regions.

In the S-IJA condition, results showed for that: (i) Synchronization strength of Regions A, C, and left hemisphere had significantly positive correlations with family income (*r* ≥ 0.28, *p* < 0.05); and (ii) Synchronization strength of Region E and right hemisphere, as well as synchronization asymmetry in region-pair E-B and right-left hemisphere had significantly positive correlations with CI^ (1)^ emotional understanding ability (*r* ≥ 0.28, *p* < 0.05).

In the S-RJA condition, it was revealed that: (i) Synchronization asymmetry in region-pair D-A was positively correlated with Raven’s reasoning ability (*r* = 0.28, *p* = 0.03); and (ii) Synchronization strength of Region D, right hemisphere and whole regions were also positively correlated with CI^ (1)^ expression recognition ability (*r* = 0.28, *p* = 0.03).

In the T-IJA condition, results verified that synchronization strength of Regions B and F were positively correlated with CI^ (1)^ emotional understanding ability (*r* ≥ 0.30, *p* < 0.05). In the T-RJA condition, it was confirmed that synchronization asymmetry in region-pair F-C was negatively correlated with Raven’s reasoning ability (*r* = −0.28, *p* = 0.03). There were no correlations between other pairs among behavioral measures and synchronization strength and asymmetry (*p’s* > 0.05).

## Discussion

Previous studies showed that disrupted synchronization appears to be a notable characteristic of neurodevelopmental disorder and contributes to several neurological symptoms, including ADHD, autism, and Schizophrenia. On the other hand, the prefrontal cortex plays a crucial role in executive functions and emotion regulation. However, little is known whether there is decreased prefrontal brain synchronization in left-behind children. On the other hand, previous studies showed that left-behind children might have deficits in social cognition. This study aimed to explore whether there is decreased prefrontal synchronization during joint attention in left-behind children. By collecting task-related fNIRS imaging data from the prefrontal cortex as well as behavioral measures (associated with family income, intelligence, language, and social-emotional abilities), we found that non-left-behind children evoked significantly higher synchronization strength and asymmetry during joint attention in some brain regions (e.g., Region F, right middle frontal gyrus) than left-behind children. Moreover, for left-behind children, the interaction with familiar persons initiated higher synchronization strength and asymmetry in some brain regions (e.g., Region E, right middle frontal gyrus) than unfamiliar persons. Also, prefrontal synchronization strength and asymmetry during joint attention were significantly correlative with these behavioral measures. These findings support the deficits of social cognition in left-behind children may be predicted by decreased prefrontal synchronization strength and asymmetry during joint attention.

The prefrontal cortex engages in various cognitive functions such as inhibition, and working memory, and cognitive flexibility. Through its inhibitory pathway to the amygdala, the prefrontal cortex involves in emotional cognition and regulation (Johnstone et al., 2007). We interestingly found for left-behind children that there were decreased synchronization strength and asymmetry during joint attention in brain regions (e.g., bilateral middle frontal gyrus and inferior frontal gyrus) located in the right prefrontal cortex. Such a result supports that decreased right prefrontal synchronization strength and asymmetry during joint attention appear to be vulnerability factors influencing the emotional and social cognition and regulation processing in left-behind children. This finding is consistent with previous results that: (i) Right-prefrontal cortical activity was associated with negative affect, withdrawal motivation, experienced anger and aggression, and emotion regulation (Eddie and Jonathan, 2001; Lee et al., 2012); (ii) Right-hemispheric prefrontal asymmetry influenced affective recovery and cognitive performance following a failure experience (Haehl et al., 2020); (iii) A decreased activation was found in Schizophrenia patients during fear, mixed fear and mixed happy processing in the right prefrontal cortex (Szabó et al., 2017); and (iv) Right prefrontal cortex contributed to the psychopathology of young children with autism at the functional network architecture level and at the functional lobe-connectivity level, respectively (Li and Yu, 2016). As far as we know, this is the first time to suggest that decreased right prefrontal synchronization strength and asymmetry during joint attention can be considered as vulnerability factors for the development of left-behind children. Based on this inference, appropriate intervention tools will deserve to be investigated in future research.

Previous studies have verified that the parent-child separation contributes to abnormal alterations of brain structure, including gray matter volume, fractional anisotropy, and network efficiency. In particular, these accelerated or decelerated structure changes involving emotion circuits may contribute to potential risks of psychiatric disorders across the lifespan. However, these studies consider the resting-state brain structure and network only (task-free case). Our results are task-related and bring some new insights in the understanding of the neural mechanism in left-behind children. For instance, we observed the emergence of decreased synchronization strength and asymmetry in the right middle frontal gyrus during joint attention in left-behind children. In addition, the interaction with familiar and unfamiliar persons evoked different synchronization strength and asymmetry.

Previous behavioral studies have shown that left-behind children might have deficits in social and emotional development, self-awareness, and mental health. As shown above, for left-behind children, decreased right prefrontal synchronization strength and asymmetry during joint attention contributes to worse temporal coordination and consistency, implying lower efficiency of executive functions and thus worse performance in social cognition tests. Such an inference is verified and consistent with the behavioral finding that non-left-behind children had better social-emotional ability (in the Emotional Comprehension Task) than left-behind children. However, by controlling the effect of family income, we also found that there were no differences for other tests, including the Combined Raven Test (an intelligence test) and PPVT (a Chinese receptive language task). This finding supports that the main differences between left-behind and non-left-behind children might be more relative with emotional and social cognition, instead of other cognitive abilities such as intelligence and language. The hypothesis is consistent with previous behavioral studies that left-behind children had potential risks to suffer from social-emotional problems than their counterparts (Zhao and Yu, 2016; Wu et al., 2019). Remarkably, these behavioral measures were significantly correlative with prefrontal synchronization strength and asymmetry in left and right hemispheres, and different brain regions (Regions A-F). This consistency of widespread correlations partially verifies the reliability of the suggested method.

The past several years have witnessed vast advances in synchronization [see a review (Pikovsky et al., 2001)]. Several synchronization estimation methods have been developed (Mahdi et al., 2014). Most of them are based on bivariate measures (e.g., cross correlation, coherence, phase-locking value, and mutual information) (Mahdi et al., 2014). However, such the bivariate analysis cannot explain the overall covariance information contained in a multichannel ensemble for a typical experimental setting. The relationship and difference between bi- and multi-channel analysis were discussed as well (Mahdi et al., 2014). To cope with the drawback of the bivariate technique, a few multivariate analysis methods have been proposed, such as the Omega complexity (based on principal component analysis), the S-estimator (derived from state space analysis), and the S-Renyi estimator (a modified version of S-estimator). Their clinical utilities have been confirmed in the evaluation of neural disorders, such as autism (Jia et al., 2018), mild spastic diplegia (Fei et al., 2016), Schizophrenia (Irisawa et al., 2006), and Alzheimer’s disease (Czigler et al., 2008). However, the Omega complexity is a biased estimation, while S-estimator does not fully consider the effects of random and pseudotrace components and thus its computational accuracy needs to be further improved. The S-Renyi method is a modified S-estimator version using the Renyi entropy, and its advantage is based on its stronger robustness, lower noise sensitivity, and flexibility (dependent on the parameter α) (Biey and Righero, 2009). More importantly, it has been confirmed that S-Renyi is more suitable for non-linear signal analysis than Omega complexity and S-estimator (Mahdi et al., 2014), because most of neural signals and systems are non-linear.

The emergence of millions of left-behind children has become a common issue in developing countries (including China), and might initiate serious economic, social, and even political issues in the future. Early deprivation of parental care may be considered as an early-life stress (Mueller et al., 2010) and a kind of adverse childhood experiences (McLaughlin et al., 2014, 2017; Teicher et al., 2016), and thereby may contribute to unexpected abnormal changes in brain structure, function, and dynamics in left-behind children. For instance, the current study has verified the emergence of decreased synchronization strength and asymmetry in the right prefrontal cortex in left-behind children. Brain development is sculpted by experiences, particularly those occurring during early sensitive or critical periods (Teicher et al., 2016). Therefore, early intervention may play an important role in supporting the brain development of left-behind children, and prevents the damaging of brain functions in the crucial period.

Most behavioral studies on left-behind children have a large sample size [e.g., *n* = 875 in the work (He et al., 2012)], while brain imaging studies usually have a relatively small sample size. A resting-state fMRI study, for instance, recruited only 26 left-behind and 21 non-left-behind children (Zhao et al., 2016). And, in the work of Fu et al. (2019), 38 left-behind and 30 non-left-behind children were considered. According to the statistics [see (Weeks and Atlas, 2015) for an example determining the sample size of intervention for children with autism], the sample size can be calculated depending on confidence level and accuracy. Based on Weeks’s work (Weeks and Atlas, 2015), the current study requires at least *n* = 25 samples in the case of 90% confidence level and +10% accuracy. Therefore, the sample size (including 34 left-behind children and 26 non-left-behind children) of this study is moderate. Clearly, one may obtain higher confidence level by increasing the sample size.

## Conclusion

Both behavioral measures and brain imaging data are correlative and support that left-behind children have deficits in social-emotional abilities. Decreased right prefrontal synchronization strength and asymmetry during joint attention might be vulnerability factors in the development of left-behind children. Public efforts are needed to provide more social/emotional and academic supports that prevent left-behind children from damaging right prefrontal synchronization strength and asymmetry, especially at very early stages of development. In a future research, we will increase the number of subjects to give additional strength to our findings, even though the sample size of this study is moderate. Also, we will explore effective intervention methods and expect to evaluate the intervention results by measuring behavioral performance scores as well as right prefrontal synchronization strength and asymmetry during joint attention.

## Data Availability Statement

The raw data supporting the conclusions of this article will be made available by the authors, without undue reservation.

## Ethics Statement

The studies involving human participants were reviewed and approved by all study procedures and research methods were carried out in accordance with the Declaration of Helsinki (1964) by the World Medical Association concerning human experimentation and were approved by the Research Ethics Committee at Southeast University. Written informed consent to participate in this study was provided by the participants’ legal guardian/next of kin.

## Author Contributions

KD, CL, and DY developed the idea for the study. KD and CL collected the data. KD and HW did the analyses. KD, HW, FL, and DY wrote the manuscript. All authors contributed to the article and approved the submitted version.

## Conflict of Interest

The authors declare that the research was conducted in the absence of any commercial or financial relationships that could be construed as a potential conflict of interest.

## Publisher’s Note

All claims expressed in this article are solely those of the authors and do not necessarily represent those of their affiliated organizations, or those of the publisher, the editors and the reviewers. Any product that may be evaluated in this article, or claim that may be made by its manufacturer, is not guaranteed or endorsed by the publisher.
